# Punicalagin Protects Human Retinal Pigment Epithelium Cells from Ultraviolet Radiation-Induced Oxidative Damage by Activating Nrf2/HO-1 Signaling Pathway and Reducing Apoptosis

**DOI:** 10.3390/antiox9060473

**Published:** 2020-06-02

**Authors:** Maria Elisabetta Clementi, Beatrice Sampaolese, Francesca Sciandra, Giuseppe Tringali

**Affiliations:** 1 National Research Council (CNR), Istituto di Scienze e Tecnologie Chimiche “Giulio Natta” (SCITEC)—c/o Istituto di Biochimica e Biochimica Clinica Università Cattolica del Sacro Cuore, Largo F. Vito 1, 00168 Rome, Italy; elisabetta.clementi@scitec.cnr.it (M.E.C.); beatrice.sampaolese@scitec.cnr.it (B.S.); francesca.sciandra@scitec.cnr.it (F.S.); 2 Pharmacology Section, Department of Health Care Surveillance and Bioethics, Fondazione Policlinico Universitario A. Gemelli IRCCS, Roma—Università Cattolica del Sacro Cuore, Largo F. Vito 1, 00168 Rome, Italy

**Keywords:** punicalagin, ARPE-19 (human-RPE cell line), nuclear factor erythroid 2-related factor (Nrf2), heme oxygenase-1 (HO-1), NADPH quinone dehydrogenase-1 (NQO1), apoptosis

## Abstract

The oxidative damage of the retinal pigment epithelium (RPE) is the early event that underlies the pathogenesis of maculopathies. Numerous studies have shown that punicalagin (PUN), a polyphenol present in pomegranate, can protect several cell types from oxidative stress. Our study aims to establish if PUN protects RPE from UV radiation-induced oxidative damage. We used an experimental model which involves the use of a human-RPE cell line (ARPE-19) exposed to UV-A radiation for 1, 3, and 5 h. ARPE-19 cells were pre-treated with PUN (24 h) followed by UV-A irradiation; controls were treated identically, except for UV-A. Effects of pre-treatment with PUN on cell viability, intracellular reactive oxygen species ROS levels, modulation of Nrf2 and its antioxidant target genes, and finally apoptosis were examined. We found that pre-treatment with PUN: (1) antagonized the decrease in cell viability and reduced high levels of ROS associated with UV-A-induced oxidative stress; (2) activated Nrf2 signaling pathway by promoting Nrf2 nuclear translocation and upregulating its downstream antioxidant target genes (HO-1 and NQO1); (3) induced an anti-apoptotic effect by decreasing Bax/Bcl-2 ratio. These findings provide the first evidence that PUN can prevent UV-A-induced oxidative damage in RPE, offering itself as a possible antioxidant agent capable of contrasting degenerative eye diseases.

## 1. Introduction

The retinal pigment epithelium (RPE), a monolayer of pigmented cells located between vessels of the choriocapillaris and light-sensitive outer segments of the photoreceptors, is a critical layer that provides trophic support to the photoreceptors [1,2]. It is therefore not surprising that RPE integrity is important for health and functionality of the choroid and retina, as well as the dysfunction and atrophy of the RPE are common pathological characteristics of various degenerative ocular pathologies [3,4]. Exposure to external stressors may induce in RPE an overproduction of reactive oxygen species (ROS) with a switch from physiological to pathological conditions [5,6]. In fact, an imbalance between the production and neutralization of ROS by antioxidant systems is associated with chronic photo-oxidative stress, which plays a key role in the pathogenesis of many age-related degenerative diseases, as well as the age-related macular degeneration (AMD) [7]. In this context, the use of dietary integrators that may oppose oxidative stress seems to be appropriate in the prophylaxis and/or therapy of degenerative diseases of the eye. This concept is based on preclinical and clinical studies that showed that an adequate supply of antioxidants, particularly of natural origin, has the potential to promote health and to reduce the risk of chronic eye diseases, including AMD and cataracts [8,9].

Scientific evidence shows that pomegranate juice contains higher antioxidants levels than red wine, green tea, and other fruit juices, such as cranberry or blueberry [10,11]. Antioxidant properties and the free-radical-scavenging activity of pomegranate have been linked to its high polyphenol content [12]. Of the polyphenols found in pomegranates, punicalagin (2,3-hexahydroxydiphenoyl-gallagyl-d-glucose; PUN) is the major bioactive component and it is responsible for >50% of the antioxidant activity of pomegranate juice [12]. Indeed, a literature review highlights that PUN is able to inhibit the oxidative stress induced by different stimuli in various experimental in vitro and in vivo models [13,14,15]. In this regard, our group has previously reported that PUN increases neuronal resistance to hydrogen peroxide-induced toxicity by inhibition, both oxidative cellular potential and apoptotic signals [16].

In light of the above observations, it is easy to postulate that high ROS levels in RPE are directly associated with degenerative ocular pathologies and that nutritional supplementation with PUN could protect the eyes from systemic oxidative damage. Therefore, to test this hypothesis, we investigated the antioxidant effects of the pre-treatment with PUN on a human-RPE cell line (ARPE-19) after short and long-time UVA-radiation induced oxidative damage, in an experimental paradigm developed and validated previously by our group [6].

Within this conceptual framework, in the present study, we also explored: (i) a possible functional link between PUN and oxidative damage mediated by nuclear factor erythroid-2-related factor 2 (Nrf2), a transcription factor that plays key roles in retinal antioxidant and detoxification responses [17,18]. (ii) If PUN induces the activation of phase-2 antioxidative enzymes [NADPH quinone dehydrogenase-1 (NQO1) and heme oxygenase-1 (HO-1)], via the Nrf2 pathway, hypothesizing a mechanism of action that supports the ability of the PUN to protect RPE from UV-A rays-induced oxidative stress [19,20]. (iii) Finally, if the hypothesized cytoprotective action of the PUN is linked to a modification of the balance Bax/Bcl-2, positive and negative regulator of cell death, respectively [21].

## 2. Materials and Methods

### 2.1. Cell Culture Conditions and UV Exposure

The human RPE cell line (ARPE-19), was purchased from the American Type Cell Culture (ATCC - CRL-2302, Manassas, VA, USA). The cells were grown in 50/50 Ham’s F12/Dulbecco’s modified Eagle’s Medium, containing 15% fetal bovine serum and penicillin/streptomycin (100 U/mL) and incubated in a humidified environment at 37 °C with 5% CO_2_. After one week, the cells were seeded in cell culture dishes (Ø 35 mm) or 96-well plates, and when reached confluence, were treated according to the experimental design. 

Irradiation was performed using a UV lamp (Vilber Lourmat VL-62C Power 6W, Marne-La-Vallée Cedex 3, France) emitting light with wavelengths at 365 nm. Exposure of cells to UV-A radiation was executed 10 cm from the source with an intensity of approximately 0.06 J/cm^2^/s. During UV-A exposure, only a thin layer of medium (2 mL or 200 µL respectively in Petri-dish or well) was placed on the cells to reduce the possible interferences with radiation. Cells were irradiated for 1, 3, or 5 h in the presence or absence of 24-h pre-treatment with PUN. PUN powder (Sigma-Aldrich, St. Louis, MO, USA; link to PubChem: https://pubchem.ncbi.nlm.nih.gov/compound/Punicalagin) was dissolved in ethanol and successively diluted to appropriate concentrations in phosphate buffer. All solutions were freshly prepared before each experiment.

### 2.2. Cell Viability 

ARPE19 cells were seeded in 96-well plates at a density of 1 × 10^4^ cells/well. Medium- or PUN-pre-treated (10 μM) cells (for 24 h) were exposed subsequently at UV-A radiations for 1, 3, or 5 h. 

Cell survival was evaluated by MTS assay (Promega srl, Padova, Italy), in accordance with the manufacturer’s directions. At the end of the experiment, MTS reagent was added to cells culture where it was converted to a water-soluble form of formazan. The quantity of formazan released into the culture supernatant was measured by a spectrophotometer (PerkinElmer Inc., Waltham, MA, USA) at a wavelength of 490 nm. The quantity of formazan released into the culture supernatant was directly proportional to the number of living cells. Results were expressed as the percentage of cell viability relative to the untreated control. 

To examine the cytotoxicity of PUN, the cells were treated with or without PUN at different concentrations (1, 5, 10, and 20 µM) for 24 h. PUN was not toxic under the tested experimental conditions. 

The morphological features of ARPE-19 cells exposed to treatments at different times were analyzed and photographed by phase-contrast microscopy (TE300-Eclipse-microscope; Nikon Corporation, Tokyo, Japan) at a magnification of ×20.

### 2.3. Detection of ROS

The production of intracellular ROS was measured using a 2′,7′-dichlorofluorescein diacetate (DCF-DA)-cellular ROS detection assay kit (Abcam, Cambridge, UK). Briefly, ARPE-19 cells were cultured in 96-well microplate (25,000 cells per well) and treated with different conditions. After irradiation, the cells were treated with DCF-DA according to the manufacturer’s protocol. DCF-DA is initially a non-fluorescent compound, which is oxidized in presence of ROS into DCF, a highly fluorescent compound. Fluorescence was quantified using a CytoFluor multi-well plate reader (Victor3-Wallac-1420; PerkinElmer, Waltham, MA, USA), at excitation/emission 485/538 nm. ROS production was expressed as fluorescence intensity and presented as a percentage relative to the untreated control. 

### 2.4. RNA Isolation and RT-PCR

Total RNA from cultured cells was extracted using a SV total RNA isolation system (Promega) kit. Spectrophotometric reading at 280 and 260 nm evaluated RNA concentration. Total RNA (1 µg) was converted to cDNA using the high-capacity cDNA reverse transcription kit (Applied Biosystems, Waltham, MA, USA). The cDNA was amplified with specific primers using PowerUp Sybr Green master mix (Applied Biosystems) in an Applied Biosystems 7500 fast real-time PCR instrument. Primers used for the evaluation of gene expression are reported in Table 1. Relative mRNA levels were calculated using the threshold cycles (CTs) normalized to values measured for GAPDH in the same samples.

### 2.5. Quantification of Intracellular Levels and Nuclear Activation of Nrf2

Intracellular Nrf-2 levels were determined by colorimetric cell-based ELISA kit (LSBio, LifeSpan Biosciences; Seattle, WA, USA). Briefly, the ARPE-19 cell cultures were seeded at a density of 30,000 cells/well in a 96-well plate. At the end of the experiments, the cells were fixed with 4% formaldehyde. Successively, quenching buffer, blocking buffer, and primary antibodies (Anti-Nrf2, a rabbit polyclonal antibody, and anti-GAPDH, a mouse monoclonal antibody, used as internal positive control) were added in sequence to the cells and finally the plate was incubated at 4 °C overnight. Following the addition of the secondary antibody conjugated to the peroxidase, the samples were read with a microplate reader to OD at 450 nm. The results were normalized as OD450 of the ratio Nrf-2/GAPDH and expressed as a percentage relative to the untreated control.

Nuclear activation of Nrf2 in ARPE-19 cells was determined after the experimental treatments, as follows. Nuclear extract preparation from ARPE-19 cells was performed using the nuclear extraction kit (# ab113474-Abcam) following the manufacturer’s instructions. The concentration of proteins in each sample was normalized on total protein content in the cell pellet using the Bradford assay. Subsequently, 20 µg of nuclear protein was added to the 96-well plate provided with Nrf2 transcription factor assay kit (# ab207223-Abcam), according the manufacturer’s instructions. Active Nrf2 present in the nuclear extract specifically binds to the oligonucleotide present in the wells. An HRP-conjugated secondary antibody provides a sensitive colorimetric readout at OD 450 nm. The results were expressed as a percentage relative to the untreated control.

### 2.6. Bax and Bcl-2 Detection 

The detection of Bax and Bcl-2 proteins was performed by colorimetric cell-based ELISA kits (Assay Biotechnology, Sunnyvale, CA, USA). In brief, the cells were seeded at 3 × 10^4^ cells/well in a 96-well plate and treated according to the experimental design; at the end of the experiments, the cells were fixed in 4% formaldehyde. Successively, quenching buffer, blocking buffer, and primary antibodies (rabbit polyclonal anti-Bax, rabbit polyclonal anti-Bcl-2, and mouse monoclonal anti-GAPDH) were added in sequence. After incubation at 4 °C overnight, the secondary antibodies conjugated to peroxidase were added (HRP-conjugated anti-rabbit IgG for Bax and Bcl-2; HRP-conjugated anti-mouse IgG for GADPH) and subsequently, the samples were read with a microplate reader at 450 nm. All values obtained, normalized to GAPDH OD450, were expressed as a percentage relative to the untreated control. 

### 2.7. Statistical Analysis

Statistical analysis and figures were generate using GraphPad Prism 5 software (GraphPad, San Diego, CA, USA). Each experiment was repeated at least two times in triplicate (unless otherwise specified). All results were presented as the mean ± SEM of (*n*) replicates per experimental group. Data were analyzed by one-way ANOVA, followed by post hoc Newman–Keuls for comparisons between group means. In experiments of cell viability, the comparison between group means was analyzed by two-way ANOVA analysis for the factors time and treatment, followed by Bonferroni’s post-test. Differences were considered statistically significant if *p* < 0.05.

## 3. Results

### 3.1. Protective Role of PUN against UV-A-Induced Oxidative Stress and Cell Death

We carried out the first series of experiments in order to confirm a possible protective effect of PUN against UVA-radiation-induced damage in RPE cells (ARPE-19). For this purpose, we used an experimental model previously developed and validated in our laboratory [6]. It allowed us to examine in ARPE-19 cells the sequence of molecular and biological events induced by prolonged and continuous treatment (until 6 h) with UV-A radiation. Therefore, in order to carry out the same experimental procedures and maintain the temporal sequence as the only experimental variable, we used a fixed-dose of PUN (10 μM) in our study. The concentration 10 μM was chosen based on the following evidence: (a) we conducted cell viability studies to evaluate the cytotoxicity of PUN in ARPE-19 for 24 h: no toxicity was found in the range 1–20 µM [Control: 100%; 1 µM PUN: 95% ± 3.7%; 5 µM PUN: 96% ± 2.8%; 10 µM PUN: 98% ± 2.4%; 20 µM PUN: 95% ± 4.1%; the results are expressed as percentage of control and represented by mean ± SEM (*n* = 3)]; (b) our recent study showed that the concentration of 10 μM of PUN was effective, but not maximal, in counteracting cellular oxidative stress. In fact, the PUN given in the range 0.5–20 μM protected the cells from oxidative damage in a concentration-dependent manner, recovering cell viability to about 85% at 10 μM [16]; (c) the scientific literature review about ellagitannins supports the hypothesis that this concentration is the most appropriate and effective in contrasting different oxidative stresses in numerous cell lines [22,23,24,25].

**Figure 1:**
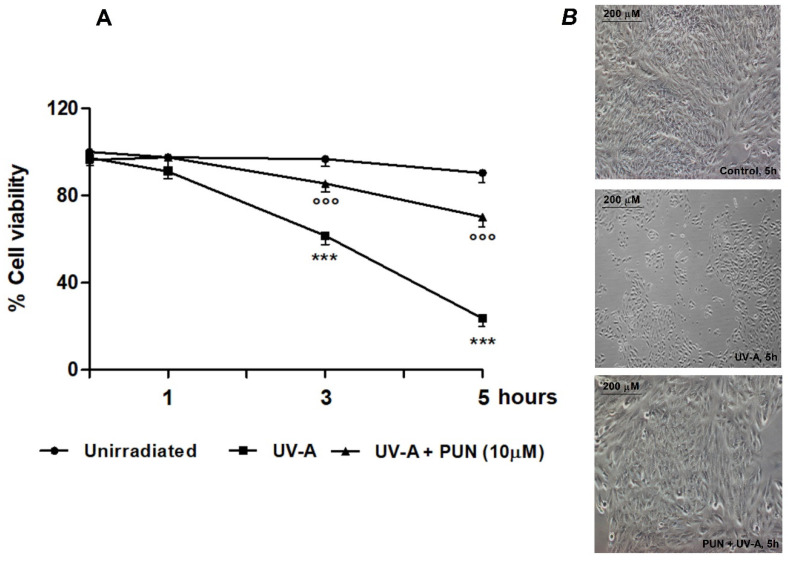
Protective effect of 10 µM Punicalagin (PUN) PUN against UV-A-induced cell death in ARPE-19 cells. (**A**) Viability of ARPE-19 cells pre-treated or not with PUN (24 h) and subsequently exposed to UV-A radiation for 1, 3, and 5 h. Data from three independent experiments are expressed as percentage viability respect to cells unirradiated at time 0 (control = 100%) and are presented as the mean ± SEM of six replicates per experimental group. *** = *p* ˂ 0.001 vs. unirradiated; °°° = *p* ˂ 0.001 vs. UV-A alone. Two-way ANOVA analysis followed by Bonferroni’s post-test was carried out. (**B**) Morphology of ARPE-19 cells observed after five hours. treatment by phase-contrast microscopy. Unirradiated cells (above); irradiated cells with UV-A (middle); cells pre-treated with 10 µM PUN before exposure to UV-A (below).

ARPE-19 cells were exposed to UV-A radiation (365 nm) at different times (1, 3, and 5 h) with and without PUN (10 µM). As expected, UV-A radiation exposure produced a time-dependent decrease in cell viability, from 1 h onward (−6.7%, −37.4%, and −74.0% viability at 1, 3, and 5 h, respectively vs. unirradiated control) (Figure 1A). Under these conditions, 24 h pre-treatment with 10 µM PUN was able to antagonize the effects of radiations (−0.1%, −11.5%, −22.4% viability at 1, 3, and 5 h, respectively vs. unirradiated control) (Figure 1A). Morphological analysis conducted in the same experimental paradigm confirmed obtained results, too (Figure 1B).

**Figure 2:**
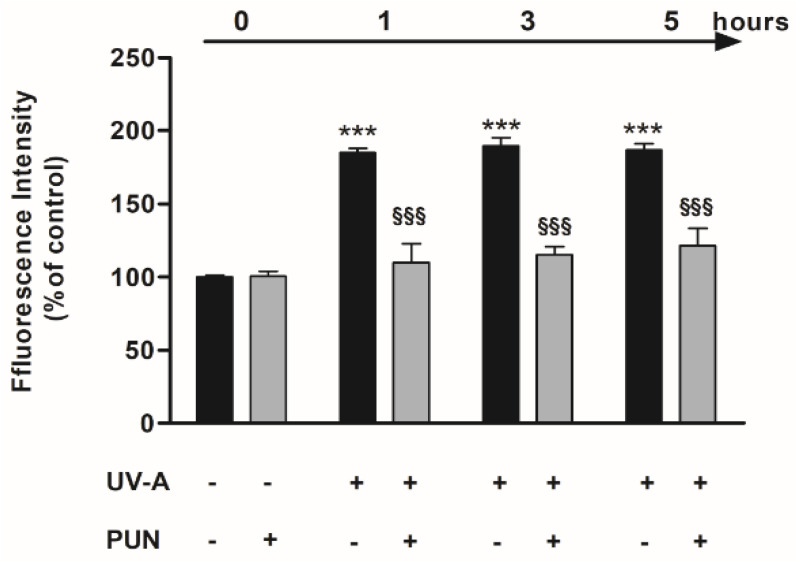
Pre-treatment with 10 µM PUN for 24 h reduces UV-A-induced increase of reactive oxygen species (ROS) levels in ARPE-19 cells. Values are expressed as a percentage relative to the control (no pre-treatment, no radiations) and are presented as mean ± SEM of data from three separate experiments; each experiment was performed in duplicate. One-way ANOVA analysis followed by post hoc Newman–Keuls was carried out. *** = *p* < 0.001 vs. control; ^§§§^ = *p* < 0.001 vs. UV-A alone at the same experimental time.

Our previous studies [6] highlighted that the reduction in cellular viability observed post-irradiation was associated with the production of ROS that generated oxidative injury. In the current study, the exposure to UV-A radiation induced a significant formation of ROS from 1 h onward, as expected. Pre-treatment (24 h) with 10 µM PUN, in the same experimental paradigm, was able to reduce ROS production induced by UV-A at all experimental times (Figure 2). PUN alone had no effect.

### 3.2. PUN Protects ARPE-19 Cells from UV Damages by Activating Nrf2/HO-1/NQO1 Signaling

**Figure 3:**
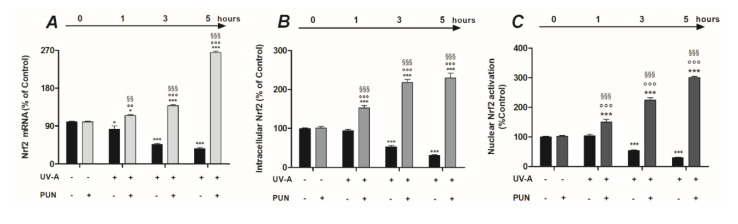
Pre-treatment with 10 µM PUN for 24 h modulates gene expression (**A**), protein synthesis (**B**), and nuclear activation (**C**) of Nrf2 in ARPE-19 cells exposed to UV-A radiation. (**A**) The levels of mRNA expression were measured by RT-PCR. Values are expressed as the mean percentage relative to unirradiated and not pre-treated cells (control 100%) ± SEM of four independent experiment performed in single. (**B**) Nrf2 protein levels were detected by colorimetric cell-based ELISA kit (see “Materials and Methods” section) and were expressed as the mean percentage relative to unirradiated and not pre-treated cells (control 100%) ± SEM of three independent experiment performed in triplicate. (**C**) To quantify Nrf2 activation in the nuclear extract, was utilized a colorimetric assay kit (see “Materials and Methods” section). All OD values were mathematically expressed as a percentage relative to unirradiated and not pre-treated cells (control 100%) ± SEM of three independent experiment performed in duplicate. Differences between mean values (shown in Figure 3) were assessed by one-way ANOVA analysis followed by post hoc Newman–Keuls. * = *p* < 0.05 and *** = *p* < 0.001 vs. control; °° = *p* < 0.01 and °°° = *p* < 0.001 vs. PUN alone; ^§§^ = *p* < 0.01 and ^§§§^ = *p* < 0.001 vs. UV-A alone at the same experimental time.

Nrf2 is a transcription factor that regulates gene expression of a wide variety of antioxidant cytoprotective enzymes and detoxification phase II through a promoter sequence known as antioxidant response element (ARE). Therefore, we were interested in exploring whether PUN was able to modulate Nrf2 in our experimental paradigm. To elucidate this, we first examined Nrf2 mRNA expression in ARPE19 cells after UV-A irradiation in presence or absence of 10 µM PUN pre-treatment. The results showed that the levels of Nrf2 expression were significantly downregulated in ARPE-19 cells exposed to UV-A radiation, from 1 h onward (−17.4%, −53.4%, −63.5% after 1, 3, and 5 h, respectively) (Figure 3A). On the contrary, pre-treatment with PUN (24 h) significantly increased the Nrf2 mRNA levels in a time-dependent manner, antagonizing the effect of UV-A on Nrf2 gene expression (Figure 3A). The subsequent studies conducted to determine the Nrf2 protein levels confirmed the findings observed in previous gene-expression experiments, under the same experimental conditions. Pre-treatment with PUN (24 h) was able to increase the Nrf2 protein levels at all irradiation time-points analyzed; conversely, the absence of a pre-treatment with PUN caused a significantly decrease of Nrf2 in the same experimental design (Figure 3B).

When exposed to an oxidative insult, Nrf2 is stabilized and translocated to the nucleus, where it activates the transcription-mediated protective responses. Therefore, to confirm our results, we examined whether PUN protective effects were linked to the nuclear accumulation of Nrf2 and to its transcriptional activity. We measured Nrf2 activation in the nuclear fraction of the cells after UV-A irradiation in presence or absence of 10 µM PUN pre-treatment. The results obtained showed that Nrf2 activation levels significantly decreased after 3 and 5 h of UV-A radiation (Figure 3C). Contrariwise, pre-treatment with PUN (24 h) not only antagonized the radiation-induced reduction, but it significantly increased the Nrf2 nuclear activation respect to unirradiated cells, at all experimental times (Figure 3C).

**Figure 4:**
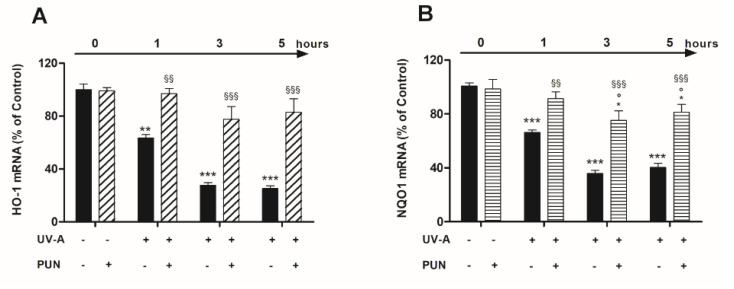
The protective effects of pre-treatment with 10 µM PUN for 24 h on UV-A-induced downregulation of Nrf2 target antioxidant genes. HO-1 (**A**) and NQO1 (**B**) mRNA expressions were assessed by RT-PCR (details in “Materials and Methods” section) at 1, 3, and 5 h after UV-A irradiation in ARPE-19 cells pre-treated and not with 10 µM PUN (24 h). Values were expressed as the mean percentage relative to unirradiated and not pre-treated cells (control 100%) ± SEM of three independent experiment performed in single. Differences between mean values were assessed by one-way ANOVA analysis followed by post hoc Newman–Keuls. ** = *p* < 0.01 and *** = *p* < 0.001 vs. control; ° = *p* < 0.05 vs. PUN alone; ^§§^ = *p* < 0.01 and ^§§§^ = *p* < 0.001 vs. UV-A alone at the same experimental time.

To strengthen the hypothesis that UV-A-induced nuclear localization of Nrf2 is followed by the acquisition of its transcriptional properties, we analyzed the mRNA levels of HO-1 and NQO1, the downstream target genes of Nrf2, in presence or absence of PUN. mRNA expression of both antioxidant genes was significantly reduced during exposure to ultraviolet radiation: the progressive reduction reached *maximum* values after three hours and remained constant thereafter (Figure 4). The pre-treatment with PUN (24 h) antagonized the UV-induced gene expression reduction of both OH-1 and NQO1. The PUN action was significant already after 1 h; but above all, it was evident at 3 and 5 h for both genes (Figure 4). 

### 3.3. PUN Modulates UV-A-Induced Bax and Bcl-2 Protein Expression

Nrf2 regulates the expression and induction of several genes in response to radiation, which results in reduced apoptosis and improved cell survival. Consequently, we performed further experiments to verify if PUN modulates intracellular levels of Bax and Bcl-2 in UV-A-treated cells. 

**Figure 5:**
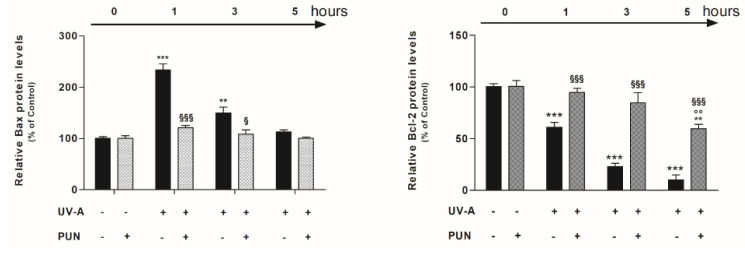
Protective effect of PUN on UV-A-induced apoptosis in ARPE-19 cells. Cells were pre-treated with 10 μM PUN and incubated for 24 h, and then exposed to UV-A radiations for 1, 3, and 5 h. Protein levels of apoptotic hallmarks, Bax (**left-panel**) and Bcl-2 (**right-panel**), were determined by colorimetric cell-based ELISA kits (details in “Materials and Methods” section). Values obtained, normalized to GAPDH, were expressed as a percentage relative to unirradiated and not pre-treated cells (control 100%). Results are from two independent experiments, each including three replicates per experimental group; they are presented as the means ± SEM. One-way ANOVA analysis followed by post hoc Newman–Keuls was carried out. ** = *p* < 0.01 and *** = *p* < 0.001 vs. control; °° = *p* < 0.01 vs. PUN alone; § = *p* < 0.05 and §§§ = *p* < 0.001 vs. UV-A alone at the same experimental time.

High protein levels of the Bax were detected after 1 and 3 h of UV-A-treatment, which dropped drastically after 5 h (Figure 5; left-panel). Contrariwise, the protein levels of Bcl-2 measured in the untreated cells (0 point), were significantly lower after UV-A-exposure in a time dependent manner (Figure 5; right-panel). In the same experimental paradigm, the pre-treatment with PUN (24 h), caused a shift in the Bax/Bcl-2 balance, promoting an anti-apoptotic action. Thus, in UV-A treated cells, PUN antagonized the high levels of Bax (maintaining protein levels similar to the untreated control), and it increased significantly the intracellular levels of Bcl-2 that are reduced by UV-A radiations (Figure 5).

## 4. Discussion

The scientific community’s interest in pomegranate fruit has grown significantly in the past decade thanks to evidence of its therapeutic effects. In particular, there is growing preclinical and clinical evidence that Pun, the main bioactive ellagitannin isolated from pomegranate, is a promising nutraceutical agent in the prevention and treatment of various pathological conditions [23]. To date, it is well known that PUN possesses antioxidant, anti-inflammatory, antidiabetic, anti-proliferative, anti-angiogenic, and anti-cancer properties. Moreover, many of the PUN’s beneficial effects have been widely related to the modulation of various and important signaling pathways and to the expression of genes responsible for the therapeutic effects mentioned above [24,25,26,27,28]. Nevertheless, the results presented in this study demonstrate for the first time that PUN has an antioxidant action on the RPE and a probable antiapoptotic effect, modulating the protein expression of Bax and Bcl2. This opens up new scenarios for its hypothetical use in the treatment of eye diseases on oxidative basis. In addition, our evidence fully introduces PUN among antioxidant natural substances able to protect RPE from oxidative stress. In this regard, there is a wide research line that describes compounds of natural origin capable of protecting RPE, via Nrf2 activation, against various oxidative stimuli (mainly H_2_O_2_) [29,30,31]. However, the fact that, in our experimental paradigm, PUN protects epithelium against UV radiation (the main natural exogenous stressor for RPE) makes it particularly noteworthy, compared to other compounds.

RPE is essential for visual function, performing important multiple functions necessary for the health and functionality of choroid and retina. However, external stress factors (smoke, radiation, pollution, age) can induce in RPE an overproduction of ROS, which causes an imbalance between the production and neutralization of free radicals causing chronic photo-oxidative stress, which underlies the pathogenesis of neurodegenerative disorder associated with maculopathy [32]. Following this line of reasoning, testing ROS-scavenging properties of PUN on RPE seemed important to us. Oxidative damage has been reproduced in vitro using a human RPE cell line (hARPE-19) exposed to an UV-A lamp radiation (range 1–5 h), mimicking the light-induced oxidative stress in the RPE in vivo. Under these experimental conditions, the cells show a framework compatible with a cytotoxicity state, characterized by high levels of ROS, which induce initially apoptosis and subsequently led to irreversible cell necrosis [6]. In the current study, we demonstrate that 24 h of pre-treatment with PUN antagonizes the radiation-induced toxicity in RPE. Indeed, PUN increases the antioxidant activity, reducing the ROS cellular levels and increasing cell viability, so that it attenuates events leading to oxidative stress-induced cell damage. This is also corroborated by the fact that pre-treatment with PUN is able to reduce oxidative stress-induced apoptotic signals, just after the first hour of UV-A rays exposure. In fact, after treatment with PUN, we observe a reversal in the relationship between Bax/Bcl-2, characterized by a reduction of Bax and an increase of Bcl-2 protein levels. Overall, these data not only confirm the antioxidant and/or anti-apoptotic properties of PUN, which have already been highlighted in other experimental models, but also open new therapeutic perspectives for its hypothetical use in ocular pathologies characterized by dysfunction of the RPE [33]. 

Because of the involvement of oxidative stress in RPE degeneration, numerous studies have been focused on the mechanisms underlying photo oxidative stress-induced RPE cell death in order to decipher the pathogenesis mechanism of maculopathies [34,35]. Keap1/Nrf2/ARE pathway signaling has been hypothesized to play an important role in the regulation of redox homeostasis. In fact, negative regulation of the Keap1/Nrf2/ARE pathway is involved in the pathogenesis of maculopathy, whereas activation of this signaling can reduce the risk of this disease [36]. In absence of oxidative stress, Keap1 represses Nrf2 activity and mediates its proteasomal degradation in the cytosol. In case of oxidative stress, Keap1 undergoes a conformational change that allows Nrf2 to translocate to the nucleus. In the nucleus, Nrf2 forms a heterodimer with small Maf proteins (sMaf) and binds to the ARE region, initiating transcription of target genes. Activation of the Nrf2/ARE-driven gene regulatory pathway puts in place a sequence of events that ultimately protect the cell from oxidative injury [37]. It is well documented that antioxidant and cytoprotective effects of Nrf2 are mediated through up-regulation of phase II antioxidant enzymes, such as HO-1 and NQO1 that counteract oxidative stress insults [38]. 

The Nrf2 signaling pathway could be taken into account also to explain the relationship between the protective effects of PUN and UV-A radiation-induced oxidative damage on RPE cells, observed in our study. Exposure to UV-A radiation causes, in ARPE-19 cells, a time-dependent, significant, and rapid reduction of both mRNA (1 h later) and protein expression (2 h later) of Nrf2 levels, reaching their lower values (−63% and −69% compared to unirradiated control, respectively) after 5 h. Twenty-four hours pre-treatment with PUN not only antagonizes the aforementioned effects, but also increases the intracellular levels of Nrf2 compared to non-irradiated control. Additionally, PUN is able to counteract UV-A-induced decrease of nuclear Nrf2 and its transcriptional activity as well as the mRNA levels of its downstream antioxidants including HO-1 and NQO1. This mechanism could also be proposed to explain the anti-apoptotic effects of PUN observed after 1 h of UV-A exposure in our experimental model. In fact, the translocation of Nrf2 can also cause an increase in the transcription of the Bcl-2 gene, which translates into a raise of Bcl-2 protein levels and a decrease of Bax, with a consequent decrease in apoptosis and increase in cell survival [39]. Despite scant information in the literature on this topic, our findings agree with current knowledge about PUN and its anti-oxidative action via Nrf2 pathways observed in other experimental models both in vivo and in vitro [24,40,41]. Interestingly, PUN has no effects on ARPE-19 cells in absence of photo-oxidative stress, such as UV-A rays. 

## 5. Conclusions

In conclusion, the present study extends our knowledge on therapeutic properties of PUN also to RPE. This is the first study that has evaluated the effectiveness of PUN as an antioxidant substance at RPE level. Our data strongly suggest that PUN and the Keap/Nrf2/ARE signaling pathway act in concert to maintain redox homeostasis, thereby protecting RPE from photo oxidative stress-induced degeneration. Furthermore, the upregulation of Nrf2, in order to maintain a redox balance and an anti-apoptotic response, is considered a promising therapeutic target for phytochemicals derived from various natural products [42]. Therefore, these findings suggest that supplementing PUN may have therapeutic value in prophylaxis and/or treatment of oxidation-associated disorders of RPE. Obviously, additional in vivo and in vitro research will be needed to fully elucidate the therapeutic efficacy and the molecular mechanisms underlying PUN protective action at the RPE level.

## Figures and Tables

**Table 1:** Primers used for the evaluation of gene expression.

Genes	Forward	Reverse	Product Length
Nrf2 (NM_006164.5)	GTCACATCGAGAGCCCAGTC	ACCATGGTAGTCTCAACCAGC	193
HO-1 (NM_002133.3)	CTGGAGGAGGAGATTGAGCG	ATGGCTGGTGTGTAGGGGAT	152
NQO1 (NM_000903.3)	GGTTTGAGCGAGTGTTCATAGG	GCAGAGAGTACATGGAGCCAC	129
GAPDH (NM_002046.7)	AACGGATTTGGTCGTATTG	GGAAGATGGTGATGGGATT	208

## References

[1] Strauss O. (2005). The retinal pigment epithelium in visual function. Physiol. Rev..

[2] Strauß O. (2016). Pharmacology of the retinal pigment epithelium, the interface between retina and body system. Eur. J. Pharmacol..

[3] Pavan B., Dalpiaz A. (2018). Retinal pigment epithelial cells as a therapeutic tool and target against retinopathies. Drug Discov. Today.

[4] Kaarniranta K., Tokarz P., Koskela A., Paterno J., Blasiak J. (2017). Autophagy regulates death of retinal pigment epithelium cells in age-related macular degeneration. Cell Biol. Toxicol..

[5] Seo S., Krebs M.P., Mao H., Jones K., Conners M., Lewin A.S. (2012). Pathological Consequences of Long-Term Mitochondrial Oxidative Stress in the Mouse Retinal Pigment Epithelium. Exp. Eye Res..

[6] Tringali G., Sampaolese B., Clementi M.E. (2016). Expression of early and late cellular damage markers by ARPE-19 cells following prolonged treatment with UV-A radiation. Mol. Med. Rep..

[7] Birben E., Sahiner U.M., Sackesen C., Erzurum S., Kalayci O. (2012). Oxidative stress and antioxidant defence. World Allergy Organ. J..

[8] Gorusupudi A., Nelson K., Bernstein P.S. (2017). The Age-Related Eye Disease 2 Study: Micronutrients in the Treatment of Macular Degeneration. Adv. Nutr..

[9] Piccardi M., Marangoni D., Minnella A., Savastano M.C., Valentini P., Ambrosio L., Capoluongo E., Maccarone R., Bisti S., Falsini B. (2012). A longitudinal follow-up study of saffron supplementation in early age-related macular degeneration: Sustained benefits to central retinal function. Evid. Based Complement. Alternat. Med..

[10] Basiri S. (2015). Evaluation of antioxidant and antiradical properties of Pomegranate (*Punica granatum* L.) seed and defatted seed extracts. J. Food Sci. Technol..

[11] Seeram N.P., Aviram M., Zhang Y., Henning S.M., Feng L., Dreher M., Heber D. (2008). Comparison of antioxidant potency of commonly consumed polyphenol-rich beverages in the United States. J. Agric. Food Chem..

[12] Gil M.I., Tomas-Barberan F.A., Hess-Pierce B., Holcroft D.M., Kader A.A. (2000). Antioxidant activity of pomegranate juice and its relationship with phenolic composition and processing. J. Agric. Food Chem..

[13] Kulkarni A.P., Mahal H.S., Kapoor S., Aradhya S.M. (2007). In vitro studies on the binding, antioxidant, and cytotoxic actions of punicalagin. J. Agric. Food Chem..

[14] Chen B., Tuuli M.G., Longtine M.S., Shin J.S., Lawrence R., Inder T., Michael Nelson D. (2012). Pomegranate juice and punicalagin attenuate oxidative stress and apoptosis in human placenta and in human placental trophoblasts. Am. J. Physiol. Endocrinol. Metab..

[15] Lin C.C., Hsu Y.F., Lin T.C., Hsu H.Y. (2001). Antioxidant and hepatoprotective effects of punicalagin and punicalin on acetaminophen-induced liver damage in rats. Phytother. Res..

[16] Clementi M.E., Pani G., Sampaolese B., Tringali G. (2018). Punicalagin reduces H2O2-induced cytotoxicity and apoptosis in PC12 cells by modulating the levels of reactive oxygen species. Nutr. Neurosci..

[17] Saito Y., Kuse Y., Inoue Y., Nakamura S., Hara H., Shimazawa M. (2018). Transient acceleration of autophagic degradation by pharmacological Nrf2 activation is important for retinal pigment epithelium cell survival. Redox Biol..

[18] Lambros M.L., Plafker S.M. (2016). Oxidative Stress and the Nrf2 Anti-Oxidant Transcription Factor in Age-Related Macular Degeneration. Adv. Exp. Med. Biol..

[19] Kleszczyński K., Zillikens D., Fischer T.W. (2016). Melatonin enhances mitochondrial ATP synthesis, reduces reactive oxygen species formation, and mediates translocation of the nuclear erythroid 2-related factor 2 resulting in activation of phase-2 antioxidant enzymes (γ-GCS, HO-1, NQO1) in ultraviolet radiation-treated normal human epidermal keratinocytes (NHEK). J. Pineal Res..

[20] Bucolo C., Drago F., Maisto R., Romano G.L., D’Agata V., Maugeri G., Giunta S. (2019). Curcumin Prevents High Glucose Damage in Retinal Pigment Epithelial Cells Through ERK1/2-mediated Activation of the Nrf2/HO-1 Pathway. J. Cell. Physiol..

[21] Edlich F. (2018). BCL-2 proteins and apoptosis: Recent insights and unknowns. Biochem. Biophys. Res. Commun..

[22] Xu L., He S., Yin P., Li D., Mei C., Yu X., Shi Y., Jiang L., Liu F. (2016). Punicalagin induces Nrf2 translocation and HO-1 expression via PI3K/Akt, protecting rat intestinal epithelial cells from oxidative stress. Int. J. Hyperth..

[23] Xu X., Guo Y., Zhao J., He S., Wang Y., Lin Y., Wang N., Liu Q. (2017). Punicalagin, a PTP1B inhibitor, induces M2c phenotype polarization via up-regulation of HO-1 in murine macrophages. Free Radic. Biol. Med..

[24] Kang B., Kim C.Y., Hwang J., Jo K., Kim S., Suh H.J., Choi H.S. (2019). Punicalagin, a Pomegranate-Derived Ellagitannin, Suppresses Obesity and Obesity-Induced Inflammatory Responses Via the Nrf2/Keap1 Signaling Pathway. Mol. Nutr. Food Res..

[25] Wang P., Peng X., Wei Z.F., Wei F.Y., Wang W., Ma W.D., Yao L.P., Fu Y.J., Zu Y.G. (2015). Geraniin Exerts Cytoprotective Effect Against Cellular Oxidative Stress by Upregulation of Nrf2-mediated Antioxidant Enzyme Expression via PI3K/AKT and ERK1/2 Pathway. Biochim. Biophys. Acta.

[26] Singh B., Singh J.P., Kaur A., Singh N. (2018). Phenolic compounds as beneficial phytochemicals in pomegranate (*Punica granatum* L.) peel: A review. Food Chem..

[27] Zhang Y., Cao Y., Chen J., Qin H., Yang L. (2019). A new possible mechanism by which punicalagin protects against liver injury induced by type 2 diabetes mellitus: Upregulation of autophagy via the Akt/FoxO3a signaling pathway. J. Agric. Food Chem..

[28] Cheng X., Yao X., Xu S., Pan J., Yu H., Bao J., Guan H., Lu R., Zahng L. (2018). Punicalagin induces senescent growth arrest in human papillary thyroid carcinoma BCPAP cells via NF-κB signaling pathway. Biomed. Pharmacother..

[29] Huang S.Y., Chang S.F., Chau S.F., Chiu S.C. (2019). The Protective Effect of Hispidin against Hydrogen Peroxide-Induced Oxidative Stress in ARPE-19 Cells via Nrf2 Signaling Pathway. Biomolecules.

[30] Sun Z.W., Chen C., Wang L., Li Y.D., Hu Z.L. (2020). S-allyl cysteine protects retinal pigment epithelium cells from hydroquinone-induced apoptosis through mitigating cellular response to oxidative stress. Eur. Rev. Med. Pharmacol. Sci..

[31] Zhao B., Wang Z., Han J., Wei G., Yi B., Li Z. (2020). Rhizoma Paridis total saponins alleviate H2O2 induced oxidative stress injury by upregulating the Nrf2 pathway. Mol. Med. Rep..

[32] Nita M., Grzybowski A. (2016). The Role of the Reactive Oxygen Species and Oxidative Stress in the Pathomechanism of the Age-Related Ocular Diseases and Other Pathologies of the Anterior and Posterior Eye Segments in Adults. Oxid. Med. Cell. Longev..

[33] Sparrrow J.R., Hicks and Hamel C.P. (2010). The retinal pigment epithelium in health and disease. Curr. Mol. Med..

[34] Hanus J., Anderson C., Wang S. (2015). RPE necroptosis in response to oxidative stress and in AMD. Ageing Res. Rev..

[35] Datta S., Cano M., Ebrahimi K., Wang L., Handa J.T. (2017). The impact of oxidative stress and inflammation on RPE degeneration in non-neovascular AMD. Prog. Retin. Eye Res..

[36] Batliwala S., Xavier C., Liu Y., Wu H., Pang I.H. (2017). Involvement of Nrf2 in Ocular Diseases. Oxid. Med. Cell. Longev..

[37] Yamamoto M., Kensler T.W., Motohashi H. (2018). The KEAP1-NRF2 system: A thiol-based sensor-effector apparatus for maintaining redox homeostasis. Physiol. Rev..

[38] Lee J.M., Li J., Johnson D.A., Stein T.D., Kraft A.D., Calkins M.J., Jakel R.J., Johnson J.A. (2005). Nrf2, a multi-organ protector?. FASEB J..

[39] Niture S.K., Jaiswal A.K. (2012). Nrf2 protein up-regulates antiapoptotic protein Bcl-2 and prevents cellular apoptosis. J. Biol. Chem..

[40] Rao F., Tian H., Li W., Hung H., Sun F. (2016). Potential role of punicalagin against oxidative stress induced testicular damage. Asian J. Androl..

[41] Xu X., Li H., Hou X., Li D., He S., Wan C., Yin P., Liu M., Liu F., Xu J. (2015). Punicalagin Induces Nrf2/HO-1 Expression via Upregulation of PI3K/AKT Pathway and Inhibits LPS-Induced Oxidative Stress in RAW264.7 Macrophages. Mediat. Inflamm..

[42] Zhao C.R., Gao Z.H., Qu X.J. (2010). Nrf2-ARE signaling pathway and natural products for cancer chemoprevention. Cancer Epidemiol..

